# Spontaneous Dislocation Clavicles

**Published:** 1874-02

**Authors:** Edgar Holden


Singular Spontaneous Dislocation of the Clavicles.—*By Edgar Holden, M.D.*—The disarticulation was upwards and forwards, and so great as to equal the diameter of the articulating extremity of the bone. These were, however, easily reduced, and at first a slight pressure was sufficient to keep them in place. Compress and strapping were resorted to in a variety of ways, but, owing to the anatomical relation of the muscles of the vicinity, without success. It is to call attention to the device which did relieve, that, if so rare a case should occur to any other member of the profession, it may at once be resorted to, that this article is written.

An artificial sternum of sole-leather was made of the size and shape of the natural one, except that its cornua were prolonged to the extent of about two inches; in other words, the inter-clavicular notch was deepened to that extent. These cornua were pulled apart to allow free motion of the sterno-cleido-mastoid muscles when the head was turned, and the new sternum was moulded while wet to the inequalities of the natural one, the cornua being bent over the ends of the clavicle. When dry this was covered with buck-skin, with straps and buckles at the cornua and lower extremity, and a broad belt made to encircle the waist below the ribs. To this belt the straps mentioned were buckled.

Counter-extension was thus secured, and the result has been highly satisfactory—the instrument proving in no great degree uncomfortable, and, after the many discouraging trials with other means, succeeding admirably in preventing any dislocation even upon unusual exertion.

